# Integrated epigenome, whole genome sequence and metabolome analyses identify novel multi-omics pathways in type 2 diabetes: a Middle Eastern study

**DOI:** 10.1186/s12916-023-03027-x

**Published:** 2023-09-08

**Authors:** Noha A. Yousri, Omar M. E. Albagha, Steven C. Hunt

**Affiliations:** 1 Genetic Medicine, Weill Cornell Medicine-Qatar, Doha, Qatar; 2 College of Health and Life Sciences, Hamad Bin Khalifa University, Doha, Qatar; 3 Computer and Systems Engineering, Alexandria University, Alexandria, Egypt

**Keywords:** Epigenetics, DNA methylation, Type 2 diabetes, Methylation quantitative trait loci, Whole genome sequence, Metabolomics, Qatar BioBank, Qatar Genome Project, Multiomics, Omics Networks, Pathways

## Abstract

**Background:**

T2D is of high prevalence in the middle east and thus studying its mechanisms is of a significant importance. Using 1026 Qatar BioBank samples, epigenetics, whole genome sequencing and metabolomics were combined to further elucidate the biological mechanisms of T2D in a population with a high prevalence of T2D.

**Methods:**

An epigenome-wide association study (EWAS) with T2D was performed using the Infinium 850K EPIC array, followed by whole genome-wide sequencing SNP-CpG association analysis (> 5.5 million SNPs) and a methylome-metabolome (CpG-metabolite) analysis of the identified T2D sites.

**Results:**

A total of 66 T2D-CpG associations were identified, including 63 novel sites in pathways of fructose and mannose metabolism, insulin signaling, galactose, starch and sucrose metabolism, and carbohydrate absorption and digestion. Whole genome SNP associations with the 66 CpGs resulted in 688 significant CpG-SNP associations comprising 22 unique CpGs (33% of the 66 CPGs) and included 181 novel pairs or pairs in novel loci. Fourteen of the loci overlapped published GWAS loci for diabetes related traits and were used to identify causal associations of HK1 and PFKFB2 with HbA1c. Methylome-metabolome analysis identified 66 significant CpG-metabolite pairs among which 61 pairs were novel. Using the identified methylome-metabolome associations, methylation QTLs, and metabolic networks, a multi-omics network was constructed which suggested a number of metabolic mechanisms underlying T2D methylated genes. 1-palmitoyl-2-oleoyl-GPE (16:0/18:1) – a triglyceride-associated metabolite, shared a common network with 13 methylated CpGs, including TXNIP, PFKFB2, OCIAD1, and BLCAP. Mannonate – a food component/plant shared a common network with 6 methylated genes, including TXNIP, BLCAP, THBS4 and PEF1, pointing to a common possible cause of methylation in those genes. A subnetwork with alanine, glutamine, urea cycle (citrulline, arginine), and 1-carboxyethylvaline linked to PFKFB2 and TXNIP revealed associations with kidney function, hypertension and triglyceride metabolism. The pathway containing STYXL1-POR was associated with a sphingosine-ceramides subnetwork associated with HDL-C and LDL-C and point to steroid perturbations in T2D.

**Conclusions:**

This study revealed several novel methylated genes in T2D, with their genomic variants and associated metabolic pathways with several implications for future clinical use of multi-omics associations in disease and for studying therapeutic targets.

**Supplementary Information:**

The online version contains supplementary material available at 10.1186/s12916-023-03027-x.

## Background

The worldwide prevalence of Type 2 diabetes (T2D) in 2021 was estimated to be 9.8% [1]. However, the prevalence in countries from the Middle East and North Africa was 18.1%. The prevalence of T2D in Qataris has been estimated to be 14–17% [2], similar to other Middle Eastern populations. Such high prevalence of T2D in Qatar compared to western populations increases the risk of diabetes complications, including cardiovascular disease, retinopathy, nephropathy and early mortality. More than 400 independent genetic loci have been found to be associated with T2D across different ethnicities and yet explain only a modest portion of the prevalence [3–5]. T2D is caused by complex interactions of multiple factors including genetic, epigenetic and environmental influences. Current evidence indicates that lifestyle and environmental factors can influence gene expression and clinical phenotype through epigenetic mechanisms such as changes in DNA methylation. Identifying the epigenetic factors driving T2D are thus important to our understanding of T2D etiology.

DNA methylation in blood or pancreatic beta cells has been associated with T2D in multiple populations [6–13]. Most used a previous version of the Infinium EPIC array utilizing 450K CpGs (rather than the newer 850K array) and involved western populations. A previous study done on Qataris (*n* = 123) demonstrated heterogeneity in methylation between Qatari and UK populations, indicating the importance of studying multiple world populations with varying prevalence of T2D [14].

Genetic variants have also been associated with methylation sites, referred to as methylation quantitative trait loci (meQTLs), and have further elucidated factors affecting methylation. For example, 4.7 million cis- and 630,000 trans-meQTL variants were identified in one study using the 450K array [15]. Using the 850K EPIC arrays, another study identified a large number of significant SNP-CpG pairs related to various features of diabetic kidney disease [16]. Those, and other previous studies, used imputed genotypes from available SNP arrays.

Circulating metabolites have also been associated with T2D and its complications [17, 18]. Since metabolite levels are influenced by both genetics [19] and epigenetics, and methylation affects gene expression which in turn affects metabolic pathways, T2D methylation would be expected to be associated with metabolites and metabolic pathways related to T2D.

This study focuses on the epigenetic associations with T2D in a large number of subjects from the Qatari population to highlight the similarities and differences in T2D methylation compared to other populations. The integration of genomics and metabolomics with epigenetics was used to better understand the biological mechanisms underlying methylation changes associated with diabetes. First, the larger Infinium 850K EPIC array was used to identify novel methylation associations with T2D using Qatari samples. Second, whole genome-wide sequencing (> 5.5M SNPs) was used to identify methylation quantitative trait loci (meQTLs) for the T2D-associated CpGs to highlight genetically driven CpGs. Third, an untargeted panel of metabolites (Metabolon) was used to identify the associations of the T2D CpGs with the metabolome. Finally, combining these results with metabolic correlations, multi-omics networks were constructed to find functional metabolic pathways connecting the methylated genes and to understand their biological relation to T2D in this high-risk Middle Eastern population.

## Methods

### Subjects

The Qatar Biobank (QBB) is Qatar's National Repository Centre for biological samples and health information [2]. T2D patients were selected based on the following criteria: a) The patient replied “Yes” to the question “Have you ever been diagnosed by a doctor as diabetic;” or b) HbA1c was ≥ 6.5); and c) The self-reported age of diabetes onset was above 30 years or missing. Controls were selected to exclude any diabetics (T2D or T1D) according to the above criteria.

Samples were delivered to Weill Cornell Medicine’s genomics core for methylation in three batches as kits became available. Subjects in cohorts 1 (454 samples after QC, 39% with T2D) and 2 (381 samples after QC, 48% with T2D) were selected randomly within disease category with a target to select approximately 40% of the total sample (actual final percent was 43%) with T2D to increase statistical power. A third cohort, Cohort 3 (191 samples after QC, 33% with T2D), was selected after the T2D EWAS analyses of cohorts 1 and 2 were completed (batch 3) and was combined with these cohorts to increase the power of the methylation-SNP (meQTL) association analyses.

### DNA methylation profiling, quality control and statistical methods

A total of 1056 blood samples were collected and profiled using an Infinium Methylation EPIC 850K beadchip (Illumina, Inc). The R package and library “minfi” were used for quality control. Samples that failed the “qc” function and sex assignment were removed. Samples or sites with a median detection *p* value < 0.05 were kept. ~ 30,000 CpG sites were removed after dropping CpGs in a SNP locus (“droploci” function). Normalization was done using “Funnorm” [20] and beta values were used for the analysis. A total of 813,660 CpGs in autosomal chromosomes, and 1026 samples remained after QC (details in Additional file 1).

For the T2D EWAS, CpGs associated with T2D in cohort 1 were tested for replication in cohort 2 and those discovered in cohort 2 were tested for replication in cohort 1. Each time, the discovery *p*-value of 5.8 × 10^–8^ and the replication *p*-value of 0.05/(#EWAS significant CpGs identified in the discovery cohort) were used. This experiment was repeated two times, with and without adjusting for BMI, and all CpGs with significant replication in both experiments were combined.

Linear regression models were used for T2D EWAS associations, with CpG as the dependent variable and T2D the independent variable. Covariates included were sex, two principal components of the cell counts measured in the lab (neutrophils, basophils, eosinophils, monocytes, and lymphocytes), plate number, batch effects, sample well position, smoking surrogate (AHRR CpG site cg05575921) and three whole genome principal components to correct for population stratification [21] and relatedness. Batch effects were determined according to groups of samples that were profiled together, and included differences in recording the gender code in some batches, which was included as a batch-gender interaction effect. Because the random selection of subjects for cohorts 1 and 2 resulted in different distributions of BMI among cases and controls, we used both BMI-adjusted and BMI-unadjusted association models. As age was found to be collinear with T2D in our cohorts, an age residual model was used for age adjustment in the T2D EWAS, where the CpGs were first regressed with age in controls and then CpGs in all samples were adjusted using the β0 (intercept) and β1 coefficients resulting from regression. Moreover, the CpGs that were identified as associated with T2D in the EWAS were further tested for association with age at a later stage where a larger set of controls was available. Only two CpGs were found to be associated with age as indicated in Additional file 2: Table S1.

Finally, pathway analysis was done using the Enrichr online tool [22, 23], where all genes of the 66 identified CpGs were submitted and KEGG pathways were selected. All KEGG results were used to assign the genes to pathways in order to discuss their biological relevance to diabetes.

### Whole genome sequence, quality control and statistical methods (methylation quantitative trait loci)

A total of 5 million variants remained for analysis after quality control (Additional file 1**)** for 1026 samples. The subjects were divided into discovery (*n* = 703) and replication cohorts (*n* = 323) according to the genomic profiling batches. For the meQTL analysis (SNP-CpG), mixed models were used and included kinship, age, sex, T2D status, BMI, two principal components of the actual cell counts (neutrophils, basophils, eosinophils, monocytes, and lymphocytes), plate number, batch effects, sample well position, smoking surrogate (AHRR CpG site cg05575921) and three whole genome principal components as covariates. This analysis was done using the “Genabel” package [24] in the R statistical package, specifically the “polygenic” and “mmscore” functions [25].

### Metabolomics, quality control and statistical methods (methylation-metabolite association analysis)

A total of 1160 serum metabolites were measured using an untargeted metabolomics platform by Metabolon Inc. in 2985 samples. Data was quality controlled by log transforming the values, removing outliers (above or below 3 standard deviations from the mean over all samples for each metabolite) and z-scoring the metabolite values. A total of 936 metabolites remained for association with T2D after quality control. For the T2D MWAS analyses, the samples were randomly divided into a discovery (*n* = 1791, 70%) and a replication cohort (*n* = 1194, 30%). Additional file 2: Table S12 shows the distribution of gender, age and BMI in the discovery and replication cohorts.

For CpG-metabolite association analyses, 708 samples from combined cohorts 1, 2 and 3, that had both metabolomics and methylation were divided by batch into two sets, and METAL software [26] was used for the meta-analysis. Associations with heterogeneity at *p* < 0.05 were excluded and corresponded to Isq > 74.

First, a T2D metabolome wide association study (MWAS) was performed to identify associations between metabolites and T2D, using linear regression models, with the metabolite as the dependent variable and T2D as the independent variable, including age, sex, BMI, and three whole genome principal components as covariates. Second, using the metabolites identified as significant from the MWAS and the CpGs significant from the T2D EWAS, CpG-metabolite associations were computed using the same model and covariates (excluding BMI) from the T2D EWAS analyses, replacing T2D status with T2D metabolites.

### Mendelian randomization

A two sample mendelian randomization (2SMR) analysis was used where CpG-HbA1c associations were computed in our cohort for CpGs that had meQTL SNP-HbA1c association statistics reported in the GWAS catalogue or Stanford Bio Bank. Linear regression models for CpG-HbA1c associations included all covariates considered in the EWAS analysis except for T2D. CpGs in HK1 and PFKFB2 were found significantly associated with HbA1c using cohort 1 and cohort 2 as discovery and replication cohorts, respectively. The causal associations were then tested for those genes with HbA1c. 2SMR statistics were computed using “mr_maxlik” (maximum likelihood) and “ivw” (inverse variance weighted) functions in “MendelianRandomization” package in R.

### Network analysis

Metabolites were corrected for all covariates mentioned previously and residuals were used for the partial correlation analysis, using “GeneNet” package in R, and utilizing a Bonferroni *p*-value threshold. Pairs of all identified CpG associations and significantly correlated metabolites were combined and visualized using Cytoscape software.

## Results

**Fig. 1:**
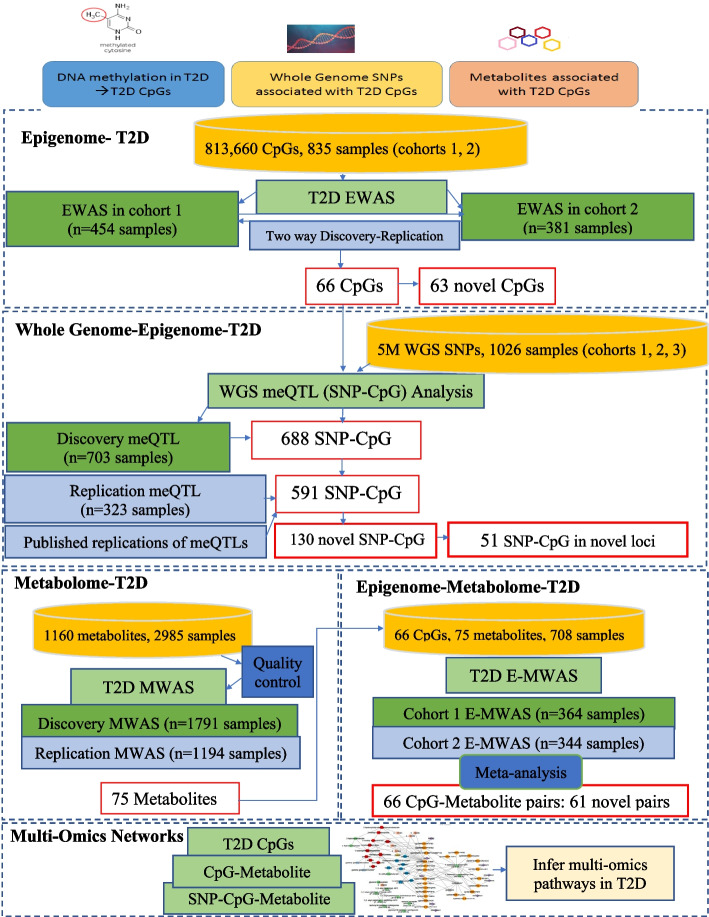
Detailed flow of the study of T2D using data from epigenetic, whole genome and metabolomics data

Figure 1 summarizes the overall study design. Three cohorts of samples were obtained from the Qatar Biobank over three years with 43.5% being T2D patients (Table 1 shows the cohort characteristics). Cohorts 1 and 2 were used for the T2D Epigenome Wide Association Study (EWAS) (*n* = 835) and cohort 3 was added to improve the statistical power of SNP-CpG association analysis (*n* = 1026). Samples from the 3 cohorts that had both metabolomics and methylation were used for the CpG-metabolite association analyses (*n* = 708).

**Table 1:** Sample characteristics for 3 cohorts selected from the Qatar Biobank

	**Whole genome- Epigenome (meQTL) analysis**					
	**T2D EWAS analysis**					
	**Cohort 1, *N* = 454**		**Cohort 2, *N* = 381**		**Cohort 3, *N* = 191**	
Variable	T2D (*N* = 176)	Nondiabetics (*N* = 278)	T2D (*N* = 183)	Nondiabetics (*N* = 198)	T2D (*N* = 63)	Nondiabetics (*N* = 128)
Age (mean ± SD)	54.4 ± 8.8	34.5 ± 10.2	51.2 ± 9.5	37.8 ± 11.4	53.4 ± 9.5	55.1 ± 12.4
BMI (mean ± SD)	32.4 ± 6.0	26.6 ± 6.0	30.8 ± 5.2	30.7 ± 5.2	32.7 ± 6.6	29.6 ± 5.6
Sex (Female %)	55.11%	50.7%	40.4%	55%	42.8%	48.4%

### 66 CpG sites in 48 genes were associated with T2D

**Fig. 2:**
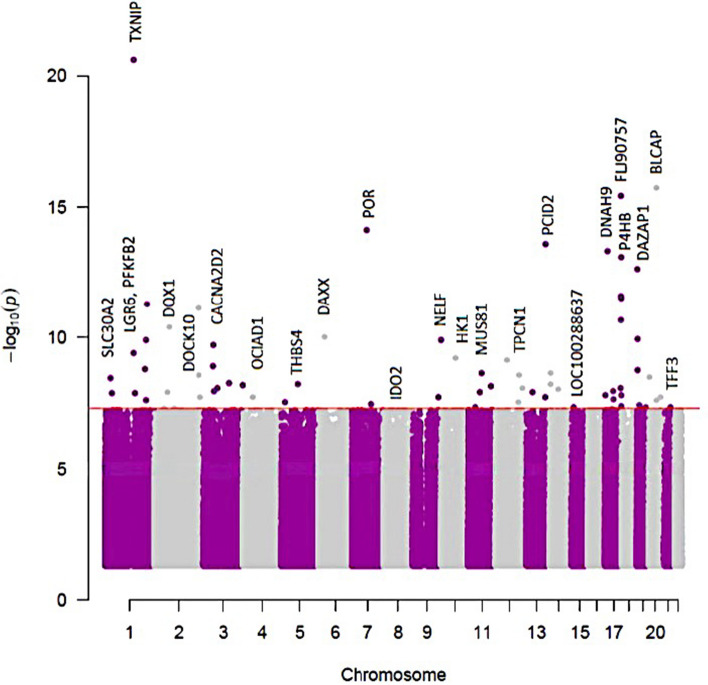
Manhattan plot of 66 CpGs associated with T2D. The red line indicates the Bonferroni p-value threshold for significance

A T2D EWAS analysis was performed using cohorts 1 and 2, in a two-way discovery and replication analysis. A total of 47 CpGs were significant at an EWAS significance threshold of *p*-value *p* < 5.8 × 10^–8^ in cohort 1 and replicated in cohort 2. An additional 10 CpGs were significant when using cohort 2 as the discovery cohort and cohort 1 as the replication cohort. When including BMI as a covariate, 33 CpG sites were identified in cohort 1 and replicated in cohort 2 and 14 CpG sites were identified as significant in cohort 2 and replicated in cohort 1. In total, 74 CpGs from models with or without adjustment for BMI were identified and replicated, out of which 66 CpGs in 48 genes had the same direction of association in both cohorts (Table 2, Additional file 2: Table S1, Fig. 2). Twenty-seven of the 66 CpGs were significant both with and without BMI adjustment. Of the 66 CpGs, only two CpGs in TXNIP were lower in T2D patients compared to nondiabetics; all other CpGs were higher in T2D compared to nondiabetics.

**Table 2:** 66 CpG sites associated with T2D from two regression models (with and without BMI adjustment)

id	Gene	**Cohort 1**				**Cohort 2**					
		**DF**	**Beta**	***p*-value**		**DF**	**Beta**	***p*-value**		**Model covariate**	**Is CpG significant in both models?**
cg19693031	TXNIP^⊥^	425	0.06	2.4E-21	*	362	0.04	7.6E-13	*	no BMI	Both
cg06291107	BLCAP	425	-0.03	1.8E-16	*	362	-0.02	3.7E-07		no BMI	Both
cg10615580	FLJ90757	425	-0.04	3.8E-16	*	362	-0.02	5.8E-06		no BMI	Both
cg01676795	POR^⊥^	425	-0.06	7.6E-15	*	362	-0.03	2.9E-06		no BMI	Both
cg20567408	PCID2	425	-0.05	2.6E-14	*	362	-0.02	3.6E-06		no BMI	Both
cg13108341	DNAH9	425	-0.08	4.8E-14	*	362	-0.05	1.2E-06		no BMI	Both
cg19420720	P4HB	425	-0.04	8.5E-14	*	362	-0.02	4.7E-06		no BMI	Both
cg00994936	DAZAP1	425	-0.03	2.3E-13	*	362	-0.02	3.7E-06		no BMI	Both
cg01219924	FLJ90757	425	-0.05	2.6E-12	*	362	-0.03	7.7E-06		no BMI	Both
cg11969813	P4HB	425	-0.05	3.0E-12	*	362	-0.03	1.9E-07		no BMI	Both
cg27094813		425	-0.04	5.1E-12	*	362	-0.03	8.4E-09	*	no BMI	Both
cg08088075		425	-0.03	7.3E-12	*	362	-0.03	3.9E-10	*	no BMI	Both
cg19707375	BAIAP2-AS1	425	-0.04	2.1E-11	*	362	-0.02	1.1E-06		no BMI	Both
cg06721411	DQX1^⊥^	425	-0.04	3.9E-11	*	362	-0.03	4.0E-08	*	no BMI	Both
cg22904406	DAXX	425	-0.04	9.4E-11	*	362	-0.02	1.0E-05		no BMI	Both
cg12973487	TCF3	425	-0.03	1.1E-10	*	362	-0.02	2.2E-06		no BMI	Both
cg14334460	NELF	413	-0.04	1.2E-10	*	361	-0.02	3.0E-05		with BMI	
cg00683922	PFKFB2	425	-0.04	1.2E-10	*	362	-0.02	2.6E-06		no BMI	Both
cg09879165	CACNA2D2	425	-0.02	2.0E-10	*	362	-0.01	6.6E-07		no BMI	Both
cg08992189	HK1	425	-0.04	6.2E-10	*	362	-0.03	1.6E-07		no BMI	Both
cg21124952		425	-0.04	7.0E-10	*	362	-0.02	3.4E-06		no BMI	
cg12761421	LGR6	413	-0.03	1.6E-09	*	361	-0.02	1.3E-05		with BMI	
cg15007470	TCF3	413	-0.04	1.8E-09	*	361	-0.02	1.5E-05		with BMI	
cg27305772	MUS81	413	-0.03	2.3E-09	*	361	-0.01	3.8E-05		with BMI	
cg06646796	DHRS2	425	-0.03	2.4E-09	*	362	-0.02	5.7E-06		no BMI	
cg15326645		413	-0.03	2.7E-09	*	361	-0.02	6.6E-06		with BMI	
cg19358608		425	-0.03	3.4E-09	*	362	-0.03	3.1E-06		no BMI	
cg01689405	SLC30A2	425	-0.03	3.6E-09	*	362	-0.02	4.7E-06		no BMI	
cg21279706	KIAA1257	425	-0.04	5.6E-09	*	362	-0.02	5.9E-06		no BMI	
cg10167677	THBS4	425	-0.03	5.9E-09	*	362	-0.03	2.0E-07		no BMI	Both
cg12350057	PPP1R3E	413	-0.05	6.0E-09	*	361	-0.03	4.4E-05		with BMI	
cg01307606		425	-0.03	6.7E-09	*	362	-0.02	1.5E-06		no BMI	Both
cg09777883		425	-0.03	7.5E-09	*	362	-0.03	2.3E-08	*	no BMI	Both
cg09029192	TNRC6C	425	-0.03	8.7E-09	*	362	-0.02	1.0E-05		no BMI	
cg20006294	FRMD4B	425	-0.04	8.8E-09	*	362	-0.02	4.2E-06		no BMI	
cg03037271		425	-0.03	1.1E-08	*	362	-0.02	3.3E-07		no BMI	
cg19225036	PBRM1	413	-0.04	1.1E-08	*	361	-0.02	2.7E-05		with BMI	
cg22896572		425	-0.04	1.2E-08	*	362	-0.04	1.0E-06		no BMI	Both
cg06555354	SPRED2	413	-0.04	1.2E-08	*	361	-0.02	4.0E-05		with BMI	
cg08110950		425	-0.02	1.2E-08	*	362	-0.02	8.4E-07		no BMI	
cg18939666	PEF1	425	-0.03	1.3E-08	*	362	-0.02	4.0E-07		no BMI	
cg11692409	SERPINF1	425	-0.03	1.6E-08	*	362	-0.02	4.7E-06		no BMI	
cg17054691	P4HB	425	-0.02	1.6E-08	*	362	-0.02	8.7E-07		no BMI	
cg13442656	NR5A1	425	-0.03	1.9E-08	*	362	-0.02	1.2E-06		no BMI	
cg00765623		425	-0.03	1.9E-08	*	362	-0.02	6.4E-06		no BMI	
cg13547913	OCIAD1	425	-0.04	2.0E-08	*	362	-0.03	8.5E-07		no BMI	
cg26236214	ARHGEF7	425	-0.04	2.0E-08	*	362	-0.03	2.4E-06		no BMI	
cg27187909	HDAC5	425	-0.03	2.2E-08	*	362	-0.02	5.0E-06		no BMI	
cg22699725	PFKFB2	413	-0.03	2.4E-08	*	361	-0.02	2.0E-05		with BMI	
cg09627709	LMTK2	425	-0.04	3.7E-08	*	362	-0.03	9.2E-06		no BMI	
cg25693597	P4HB	413	-0.03	4.4E-08	*	361	-0.02	2.3E-05		with BMI	
cg04406114	POU2F2	413	-0.03	4.6E-08	*	361	-0.02	1.3E-05		with BMI	
cg01280703	TFF3	425	-0.02	5.3E-08	*	362	-0.02	4.7E-08	*	no BMI	Both
cg12067024	S100A7A	425	-0.02	3.3E-06		362	-0.02	1.4E-08	*	no BMI	Both
cg26747273	IDO2	413	-0.05	6.1E-06		361	-0.04	5.7E-08	*	with BMI	
cg19629891	LOC100288637	413	-0.03	1.3E-05		361	-0.03	4.8E-08	*	with BMI	
cg01004980	PRKAR2A	425	-0.03	2.0E-05		362	-0.04	1.2E-09	*	no BMI	
cg27182880	LOC101929524	413	-0.02	1.2E-04		361	-0.02	3.0E-08	*	with BMI	
cg02988288	TXNIP;NBPF20;NBPF10	425	0.02	1.3E-04		362	0.02	3.9E-10	*	no BMI	Both
cg04326337	RPRD1B	425	-0.03	1.8E-04		362	-0.04	2.4E-08	*	no BMI	
cg00842231	LOC55908;DOCK6	425	-0.01	2.5E-04		362	-0.01	3.8E-08	*	no BMI	
cg00092123	KIAA1211L	425	-0.01	2.8E-04		362	-0.02	5.2E-08	*	no BMI	
cg12331557	DOCK10	413	-0.02	4.1E-04		361	-0.02	1.9E-08	*	with BMI	
cg05919951	HAUS8	413	-0.02	4.2E-04		361	-0.03	5.0E-08	*	with BMI	
cg25358033	TPCN1	425	-0.01	4.2E-04		362	-0.02	3.0E-08	*	no BMI	
cg00929203		425	-0.01	6.3E-04		362	-0.02	2.7E-09	*	no BMI	Both

^*^ Epigenome-wide (EWAS) significant. ^⊥^ indicates a previously known T2D methylated gene. Beta column indicates the effect size from regression analysis. Cohort 1 and cohort 2 were used in a 2-way discovery and replication procedure. The 66 identified CpGs were a combination of CpGs identified in cohort 1 and replicated in cohort 2 (asterisk in the cohort 1 column), and CpGs identified in cohort 2 and replicated in cohort 1 (asterisk in the cohort 2 column), using models either adjusted or unadjusted for BMI. The presented statistics were obtained from the statistical model indicated (including or excluding BMI as a covariate) when both models were significant. More details are in Additional file 2: Table S1

Three of the 66 CpGs (TXNIP, POR, and DQX1) were previously reported at an EWAS significance, and 63 CpGs are thus novel. Thirty one of the 66 identified CpGs have been previously reported to be methylated in T2D or related traits at a nominal *p*-value (*p* < 0.05), including fasting glucose, HOMA IR, HbA1c [6, 16], or BMI (Additional file 2: Table S2). An additional 15 T2D CpGs were reported to be associated with kidney function, albuminuria and kidney function decline in [16] (Additional file 2: Table S2). We also replicated T2D methylation associations [6–8, 27, 28] at a replication *p*-value in the larger cohort using the “no-BMI” model (Additional file 2: Table S3).

Five of the 48 methylated genes, namely TCF3, OCIAD1, SPRED2, DOCK10 and LMTK2 were found to be associated with T2D in the GWAS catalogue [29], (Additional file 2: Table S2). The Biobank-based Integrative Omics Studies website (BIOS QTL) [30], showed that 7 of the 66 CpGs, namely LRFN1, RP11-629O1.2, PABPC4, CD81, COL7A1, NLGN2 and HCG18 have expression-methylation associations at FDR < 0.05 (Additional file 2: Table S4**)**.

KEGG pathways containing the genes associated with the significant CpGs included fructose and mannose metabolism (PFKFB2, HK1) and insulin signaling (PPP1R3E, HK1, PRKAR2A). HK1 is linked to galactose, starch and sucrose metabolism, carbohydrate absorption and digestion, glycolysis and gluconeogenesis, and amino sugar and nucleotide sugar metabolism (Additional file 2: Table S5).

### 688 whole genome meQTLs in 27 loci were identified for T2D CpGs

**Fig. 3:**
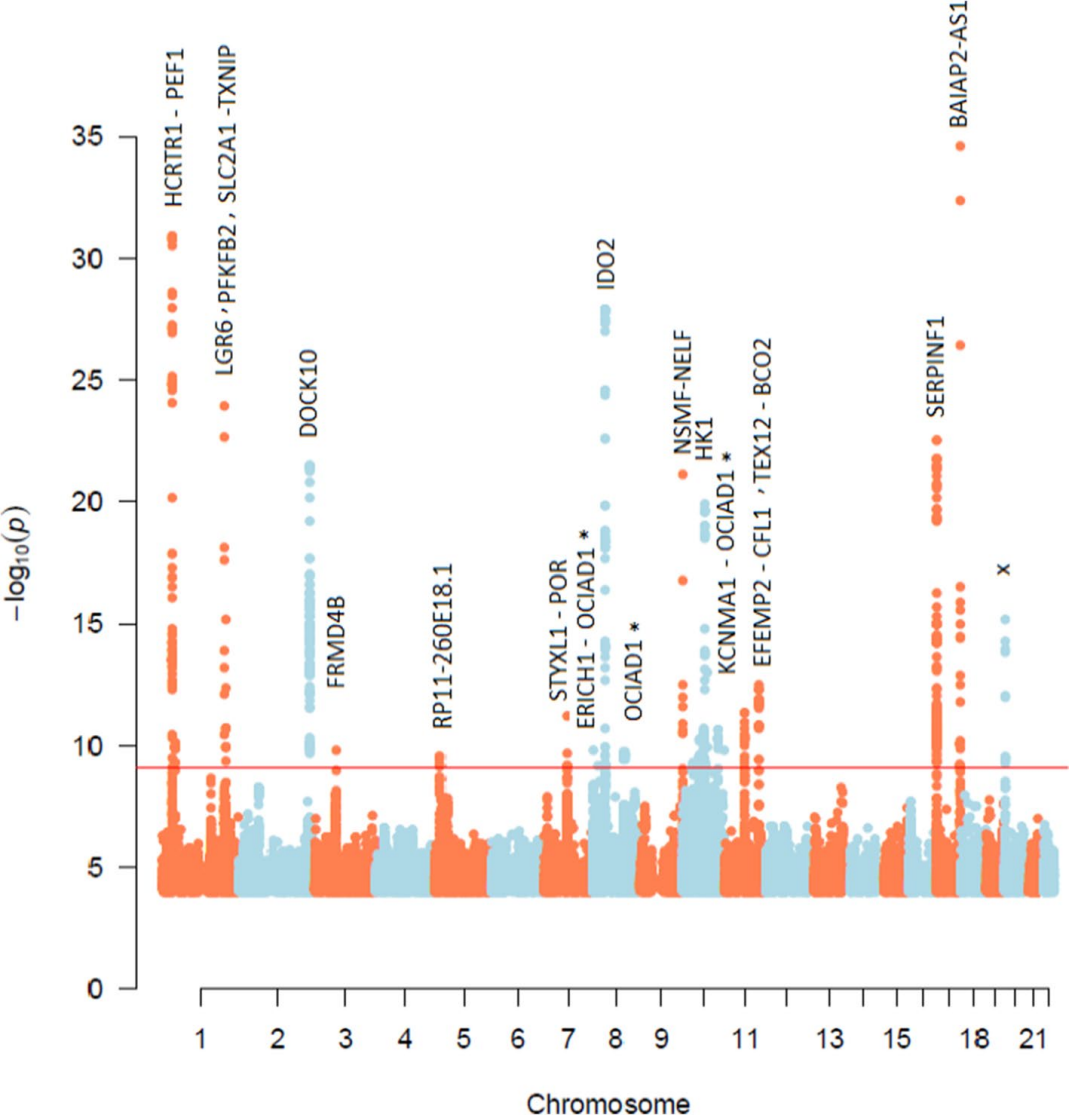
Manhattan plot of 688 CpG-SNP significant pairs. Red line is the Bonferroni p-value threshold for significance. (*p* = 8.6 × 10^–10^). “x” indicates an undefined gene or an intergenic region. “*” indicates a *trans* meQTL

The 66 significant CpG sites were tested for association with whole genome sequence SNPs (> 5.5 million SNPs) in 703 samples and tested for replication in 308 samples (see Methods). A total of 688 associations were significant in the discovery cohort at *p* < 8.7 × 10^–10^ (Fig. 3, Additional file 2: Table S6) and constituted 22 unique CpG sites that were associated with 27 unique genes, in 27 unique genic and intergenic loci. Out of 688 SNP-CpG pairs (27 loci), 222 pairs (7 loci) were either replicated at the exact SNP position or in the same locus (within 500 kb of the SNP position) in the Qatari replication cohort, and 369 pairs confirmed previous pairs or loci in Sheng et al. [16] or in BIOS QTL [30]. Collectively, 591 pairs (86% of pairs) in 19 loci (75% of loci) were either replicated in the Qatari replication cohort or confirmed in previous studies with an exact pair match or in the same locus. A total of 130 pairs were considered novel CpG-SNP associations located in previously published loci and replicated in Qataris (Additional file 2: Table S6), and 51 pairs were in novel loci, replicated in Qataris (Table 3, Additional file 2: Table S6). These loci were in TXNIP-SLC2A1, OCIAD1, and SERPINF1 genes. A total of 542 (i.e., 92% of 591) pairs were *cis* pairs (defined as less than 1 Mb distance between the SNP and the CpG; however, all identified *cis* pairs were within < 134 kb distance, with an average of 32 kb) and 49 pairs were *trans* associations (24 pairs were on the same chromosome at > 100 Mb distance, and 25 pairs were on another chromosome) in TXNIP-SLC2A1 and OCIAD1. SNPs in the 591 pairs are annotated as follows: 416 in introns, 84 intergenic, 48 in a downstream/upstream gene, 14 in 3’UTR or 5’UTR, 14 non-coding exons, 6 synonymous, 3 intron-near-splice, and 6 missense and stop-gained SNPs.

**Table 3:** Fifty-one novel SNP-CpG pairs in novel loci

rsID	chr:position	CpG	Chr^a^	Position^a^	Beta*	*p*-value	function GVS	SNP Gene	CpG Gene	cis /trans
34964576	1:43409364	cg19693031	1	145441552	94.97	1.40E-10	intron	SLC2A1	TXNIP	trans
35022307	1:43409420	cg19693031	1	145441552	94.97	1.40E-10	intron	SLC2A1	TXNIP	trans
751210	1:43410859	cg19693031	1	145441552	94.97	1.40E-10	intron	SLC2A1	TXNIP	trans
16830121	1:43412469	cg19693031	1	145441552	95.49	1.18E-10	intron	SLC2A1	TXNIP	trans
3768037	1:43412662	cg19693031	1	145441552	95.49	1.18E-10	intron	SLC2A1	TXNIP	trans
900836	1:43412727	cg19693031	1	145441552	95.49	1.18E-10	intron	SLC2A1	TXNIP	trans
7534555	1:43413319	cg19693031	1	145441552	96.61	7.73E-11	intron	SLC2A1	TXNIP	trans
7512557	1:43413324	cg19693031	1	145441552	95.49	1.18E-10	intron	SLC2A1	TXNIP	trans
7512565	1:43413361	cg19693031	1	145441552	95.49	1.18E-10	intron	SLC2A1	TXNIP	trans
710,222	1:43413653	cg19693031	1	145441552	95.49	1.18E-10	intron	SLC2A1	TXNIP	trans
61296119	1:43414046	cg19693031	1	145441552	95.49	1.18E-10	intron	SLC2A1	TXNIP	trans
74742820	1:43414369	cg19693031	1	145441552	95.49	1.18E-10	intron	SLC2A1	TXNIP	trans
57247989	1:43414370	cg19693031	1	145441552	95.49	1.18E-10	intron	SLC2A1	TXNIP	trans
75366795	1:43414447	cg19693031	1	145441552	95.49	1.18E-10	intron	SLC2A1	TXNIP	trans
112893098	1:43414563	cg19693031	1	145441552	95.49	1.18E-10	intron	SLC2A1	TXNIP	trans
2297975	1:43415516	cg19693031	1	145441552	95.82	1.07E-10	intron	SLC2A1	TXNIP	trans
79608798	1:43416158	cg19693031	1	145441552	95.49	1.18E-10	intron	SLC2A1	TXNIP	trans
4660691	1:43417150	cg19693031	1	145441552	95.49	1.18E-10	intron	SLC2A1	TXNIP	trans
12406072	1:43419737	cg19693031	1	145441552	95.49	1.18E-10	intron	SLC2A1	TXNIP	trans
113583149	1:43423955	cg19693031	1	145441552	95.49	1.18E-10	intron	SLC2A1	TXNIP	trans
11537640	1:43424519	cg19693031	1	145441552	95.27	9.15E-11	5-prime-UTR	SLC2A1	TXNIP	trans
62621848	1:43425082	cg19693031	1	145441552	95.49	1.18E-10	non-coding-exon	SLC2A1-AS1	TXNIP	trans
4306169	1:43431433	cg19693031	1	145441552	91.40	4.91E-10	intron	SLC2A1-AS1	TXNIP	trans
4,660,240	1:43433286	cg19693031	1	145441552	91.40	4.91E-10	intron	SLC2A1-AS1	TXNIP	trans
12356274	10:115290365	cg13547913	4	48831680	-61.64	1.38E-10	intergenic	none	OCIAD1	trans
2419834	10:115296495	cg13547913	4	48831680	-61.78	1.16E-10	intergenic	none	OCIAD1	trans
11196365	10:115298288	cg13547913	4	48831680	-61.78	1.16E-10	intergenic	none	OCIAD1	trans
7898594	10:115298906	cg13547913	4	48831680	-62.67	6.10E-11	intergenic	none	OCIAD1	trans
55760941	10:115298957	cg13547913	4	48831680	-62.61	6.59E-11	intergenic	none	OCIAD1	trans
1857320	10:115300285	cg13547913	4	48831680	-62.92	5.70E-11	intergenic	none	OCIAD1	trans
1857319	10:115300382	cg13547913	4	48831680	-62.99	5.27E-11	intergenic	none	OCIAD1	trans
11596601	10:115300407	cg13547913	4	48831680	-62.99	5.27E-11	intergenic	none	OCIAD1	trans
10509979	10:115301699	cg13547913	4	48831680	-63.78	2.26E-11	intergenic	none	OCIAD1	trans
4457677	10:115302658	cg13547913	4	48831680	-62.99	5.27E-11	intergenic	none	OCIAD1	trans
57919847	10:115303435	cg13547913	4	48831680	-63.78	2.26E-11	intergenic	none	OCIAD1	trans
1157916	10:115303743	cg13547913	4	48831680	-62.99	5.27E-11	intergenic	none	OCIAD1	trans
4342960	10:115304399	cg13547913	4	48831680	-62.99	5.27E-11	intergenic	none	OCIAD1	trans
11196368	10:115304614	cg13547913	4	48831680	-62.99	5.27E-11	intergenic	none	OCIAD1	trans
118011654	10:115305727	cg13547913	4	48831680	-62.99	5.27E-11	upstream-gene	none	OCIAD1	trans
4311988	10:115306261	cg13547913	4	48831680	-63.78	2.26E-11	upstream-gene	none	OCIAD1	trans
10885471	10:115307040	cg13547913	4	48831680	-62.60	6.29E-11	upstream-gene	none	OCIAD1	trans
76070905	10:115308927	cg13547913	4	48831680	-62.76	5.97E-11	upstream-gene	none	OCIAD1	trans
59096313	17:1661829	cg11692409	17	1665181	-58.43	5.30E-10	upstream-gene	none	SERPINF1	cis
62088168	17:1662296	cg11692409	17	1665181	97.30	7.39E-21	upstream-gene	none	SERPINF1	cis
7010095	8:98766461	cg13547913	4	48831680	64.95	2.46E-10	intergenic	none	OCIAD1	trans
2917964	8:98766490	cg13547913	4	48831680	64.95	2.46E-10	intergenic	none	OCIAD1	trans
7002076	8:98767981	cg13547913	4	48831680	64.95	2.46E-10	intergenic	none	OCIAD1	trans
112977944	8:98768643	cg13547913	4	48831680	65.43	1.92E-10	intergenic	none	OCIAD1	trans
12546567	8:98769090	cg13547913	4	48831680	64.90	2.55E-10	intergenic	none	OCIAD1	trans
148246392	8:98770499	cg13547913	4	48831680	64.17	3.82E-10	intergenic	none	OCIAD1	trans
185918087	8:98771524	cg13547913	4	48831680	64.20	3.34E-10	intergenic	none	OCIAD1	trans

^a^: chr and position of the CpG. *Beta column indicates the effect size from regression analysis

**Fig. 4:**
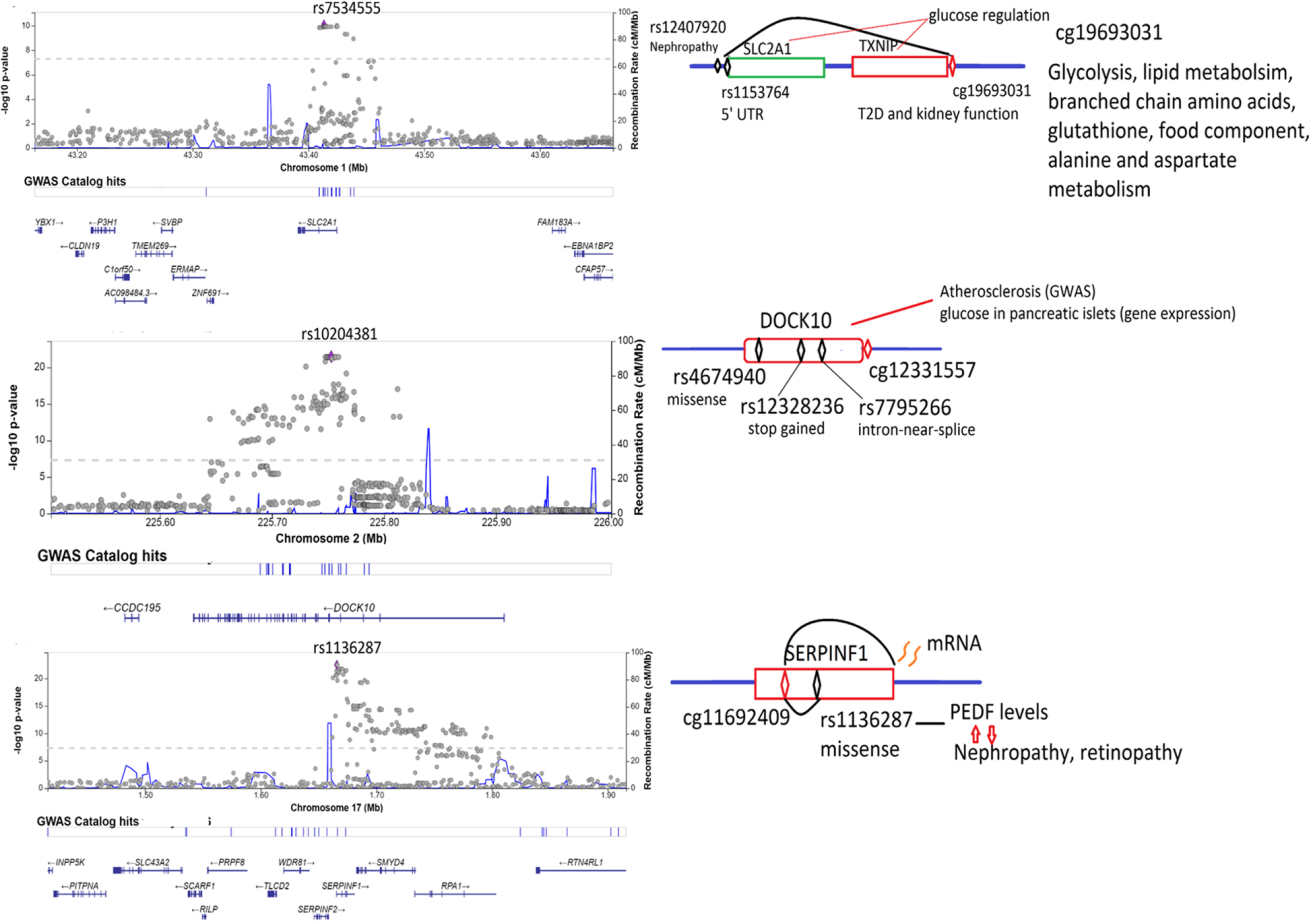
Regional plots and summary of biological functions of meQTLs in SLC2A1, DOCK10 and SERPINF1

Three meQTLs harbored nonsynonymous and 5’UTR variants, specifically in SERPINF1, DOCK10 and SLC2A1-TXNIP (Fig. 4). Four missense SNPs in the SERPINF1-SMYD4 locus were associated with cg11692409 (chromosome 17:1665181), among which rs1136287 (17:1673276) and rs1804145 (17:1674434) associated with this CpG at p = 4.0 × 10^–22^ and *p* = 5.4 × 10^–17^ respectively. The former (cg11692409-rs1136287) was also reported in [15] at *p* = 3.9 × 10^–165^ and in [16] at *p* = 3.8 × 10^–12^. Moreover, rs1136287 had been associated with pigment epithelium-derived factor (PEDF, *p* = 3.9 × 10^−35^), where PEDF was associated with diabetic nephropathy (*p* < 0.001) and sight threatening diabetic retinopathy (*p* < 0.001) [31]. Additionally, [32] showed an inverse correlation between cg11692409 and the mRNA expression of its gene (PEDF) (correlation coefficient =  − 0.38, *p* < 0.001). Another gene, DOCK10, harbored a stop-gained (rs12328236), a missense (rs4674940) and an intron-near-splice (rs77952666) variant associated with cg12331557, which were considered novel associations compared to previously known meQTLs [16]. It is worth noting that rs80176144 (~ 20 kb away from this locus) was associated with coronary artery calcified atherosclerotic plaque in T2D at *p* = 7 × 10^–6^ [33] and is ~ 50 kb away from the identified meQTL SNP. The third gene, SLC2A1, harbored a 5’UTR SNP (rs11537640) that associated with cg19693031 in TXNIP, two genes that are biologically linked [34, 35]. Located 6738 bp upstream of rs11537640, rs12407920 had been associated with diabetic nephropathy (DN) [36], whereas cg19693031, besides its association with T2D, has been associated with kidney function [37].

### meQTLs with GWAS associations and causal relationships

**Fig. 5:**
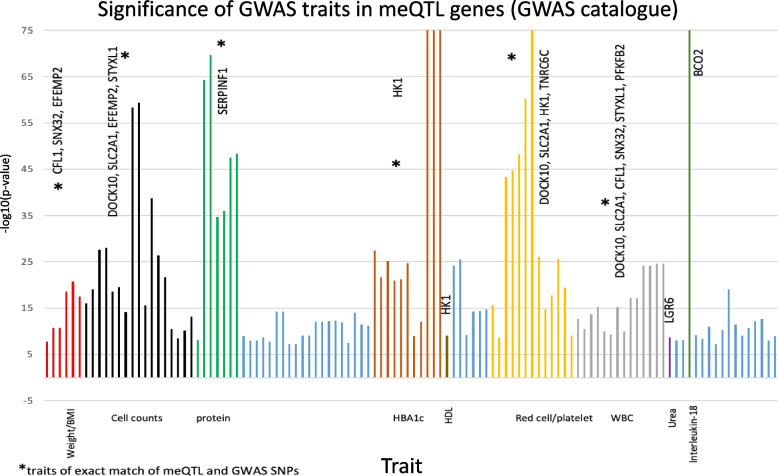
Bonferroni significant GWAS associations for traits associated with SNPs within 1kb of the meQTL SNPs. Traits are clustered according to the phenotype in > 9 clusters, indicated on the horizontal axis, that could be relevant to T2D and the blue bars are irrelevant to T2D. The marked clusters (*) have at least one exact matching meQTL and GWAS SNPs. 47 associations are exact matches, with further details shown in Additional file 2: Table S7

To investigate the biological functions of meQTL genes and identify whether meQTL SNPs are in loci that harbor GWAS associations with T2D relevant traits, we searched the GWAS catalogue [29] and the Stanford Biobank [38] for phenotypic associations with the meQTL genes. From the GWAS catalogue, 64 unique SNPs were identified at < 1kb away from meQTL SNPs, and were associated with several traits, some of which were relevant to T2D. Among those, 29 SNPs were exact matches of meQTL SNPs associated with 10 T2D CpGs (Fig. 5, Table 4, Additional file 2: Table S7). Among the most significant GWAS associations were meQTL SNPs in HK1 with HbA1c. Using the Stanford portal, we identified 20 unique SNPs located at < 1kb away from meQTL SNPs, that were associated with several traits (Table 4, Additional file 2: Table S8). Among those, 7 were exact matches of meQTL SNPs associated with 3 T2D CpGs. Both HK1 and PFKFB2 GWAS associations with HbA1c were significant in this database.

**Table 4:** Significant GWAS associations with HbA1c and exact meQTL SNP matches

GWAS catalogue results^a^						meQTL statistics	
chr:pos	**rs**	**Mapped gene**	**Reported trait**	***P* value**	**Source**	**CpG**	**p**
10:71,124,228	rs7909192	HK1	HbA1c	1E-250	Stanford portal	cg08992189	4.89E-10
10:71,098,351	rs75743765	HK1	HbA1c	1.32E-32	Stanford portal	cg08992189	7.32E-14
10:71,108,149	rs11596193	HK1	HbA1c	1.00E-09	GWAS catalogue	cg08992189	1.96E-14
1:207,250,300	rs1060286	PFKFB2	HbA1c	5.38E-10	Stanford portal	cg22699725	1.22E-10

^a^ Repeated associations with the same SNP and traits with less significance are not shown

For meQTL SNPs in HK1 and PFKFB2 that had associations with HbA1c, causal relationships of methylated genes with HbA1c as a biomarker of T2D were studied. The associations of methylation sites in those genes with HbA1c were first confirmed to be significant in our cohort. cg08992189 in HK1 was associated with HbA1c at a discovery *p*-value of *p* = 2.39 × 10^–5^ and replicated at a *p*-value of *p* = 1.6 × 10^–4^, whereas cg22699725 in PFKFB2 was associated with HbA1c at a discovery *p*-value of *p* = 2.68 × 10^–6^ and replicated at a *p*-value of *p* = 2.53 × 10^–5^. Afterwards, a two-Sample Mendelian Randomization (2SMR) analysis was conducted using CpG-SNP statistics from our cohort (meQTL results) and SNP-HbA1c statistics from the GWAS catalogue and Stanford Biobank. Table 5 shows the 2SMR results for PFKFB2 and HK1 with HbA1c (See Methods).

**Table 5:** Mendelian randomization results for associations of HK1 and PFKB2 with HbA1c

Gene	**SNP**	**CpG**	**Input**				**Two sample Mendelian Randomization**			
			Bx*	Bxse*	By*	Byse*	**Estimate**	**SE**	**CI**	***p*-value**
HK1	rs7909192	cg08992189	-100.2	16.1	0.08	0.0022	-0.00079	1.3E-04	[-0.001, -0.0005]	8.6E-10
HK1	rs75743765	cg08992189	-73.5	9.8	0.0456	0.0036	-0.0006	9.6E-05	[ -0.0008, -0.0004]	1.2E-10
HK1	rs11596193	cg08992189	-86.6	11.3	0.0277	0.004	-0.0003	6.2E-05	[-0.0004, -0.00019]	2.8E-07
HK1	rs7909192, rs75743765, rs11596193	cg08992189	~	~	~	~	-0.0007	1.8E-05	[-0.0007, -0.0006]	0†
PFKFB2	rs1060286	cg22699725	65.04	10.10	-0.013	0.002	-0.00019	4.4E-05	[-0.0002, -0.0001]	4.7E-06

^*^x is considered the exposure (CpG) obtained from meQTL statistics in our cohort and y is considered the outcome obtained from GWAS databases (GWAS catalogue or Stanford portal). Bx and Bxse stand for effect size and standard error for x, and similar symbols are used for y

^†^ Results for the 3 SNPs are from the inverse variance weighted method rather than the maximum likelihood method used for single SNPs. Heterogeneity score (I2) is 90.34007, *p* = 2.4E-20

### 66 CpG-metabolite associations were identified from T2D CpGs and T2D metabolites

**Fig. 6:**
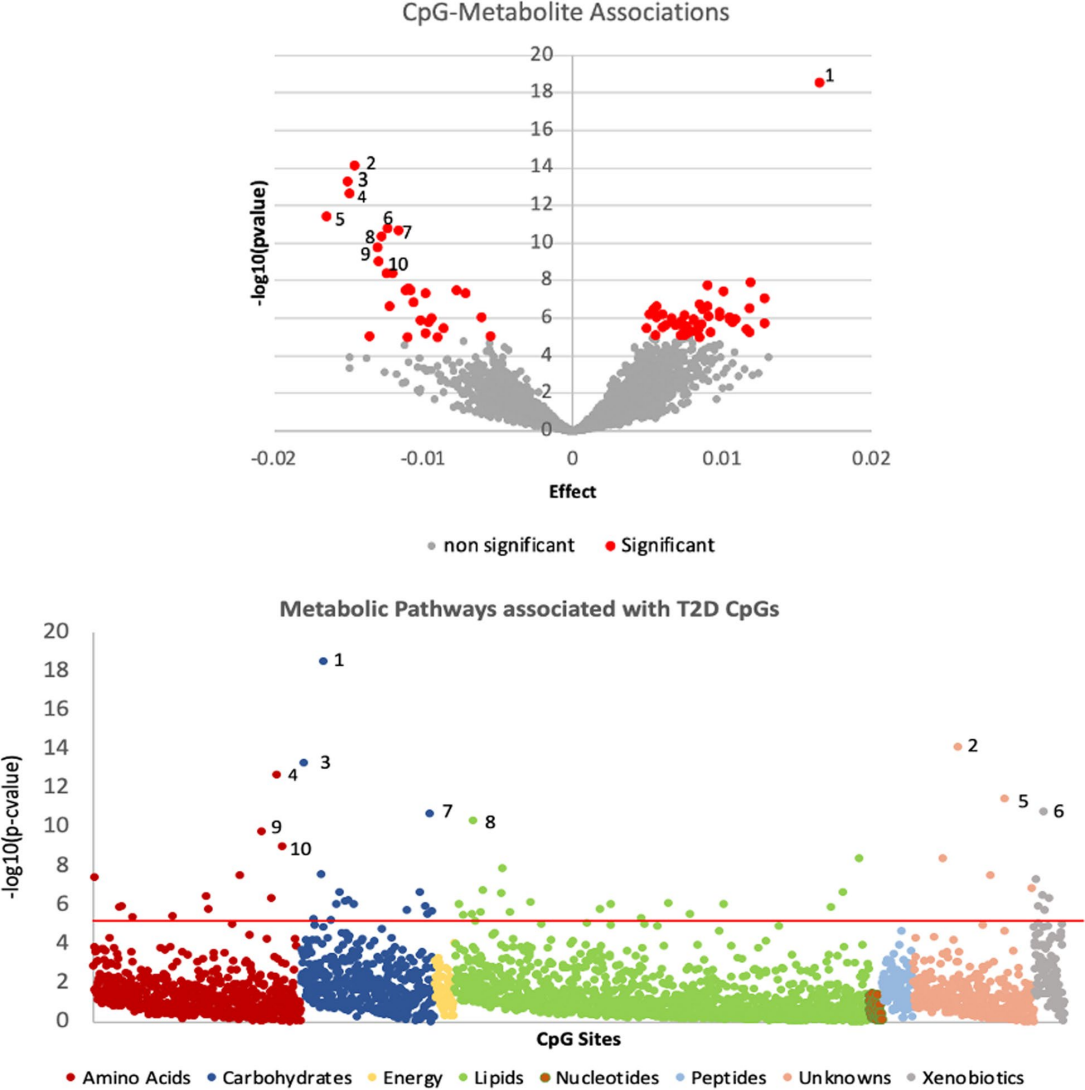
Volcano plot showing the significant CpG-metabolite associations, indicating the gene and metabolite of the top 5 significant associations (top). Metabolic pathways associated with T2D CpGs, with significant associations are above the threshold red line (bottom). The top most significant associations are labelled 1–10 and they are all TXNIP associations with (1) 1,5 AG, (2) X – 24295, (3) pyruvate, (4) 2-methyl-2-oxobutyrate, (5) X – 24334, (6) mannonate, (7) glucose, (8) 1-palmitoyl-2-arachidonoyl-GPE (16:0/20:4), (9) 2-hydroxybutyrate/2-hydroxyisobutyrate, (10) alanine

We ran a T2D metabolome wide association study (MWAS) using 2985 individuals with 936 plasma metabolites, and identified 112 metabolites that were significantly associated with T2D at a Bonferroni *p*-value threshold of (*p* < 0.05/936) in the discovery cohort (*n* = 1791), of which 75 metabolites were replicated in the replication cohort (*n* = 1194) (*p* < 0.05/112) (Additional file 2: Table S9). All 66 T2D CpGs identified in this study were tested for associations with the 75 T2D metabolites in 708 individuals. Those were divided into two cohorts of 364 and 344 individuals which were used for meta-analysis of the CpG-metabolite associations obtained from each. We identified 77 significant CpG-metabolite associations (*p* < 0.05/66*75), out of which 11 associations were removed due to a high heterogeneity, resulting in 66 significant associations in 24 unique CpGs (19 genes) and 25 unique metabolites (Table 6, Fig. 6, Additional file 2: Table S10). Of these, 61 were considered novel associations.

**Table 6:** 66 significant CpG-metabolite associations sorted by gene and significance

CpG	Gene	Metabolite	Pathway	Subpathway	Effect	*P*-value
cg19693031	TXNIP	1,5-anhydroglucitol (1,5-AG) ^a^	Carbohydrate	Glycolysis, Gluconeogenesis, Pyruvate	0.02	2.93E-19
cg19693031	TXNIP	pyruvate	Carbohydrate	Glycolysis, Gluconeogenesis, Pyruvate	-0.02	4.94E-14
cg19693031	TXNIP	Glucose^a^	Carbohydrate	Glycolysis, Gluconeogenesis, Pyruvate	-0.01	2.09E-11
cg02988288	TXNIP;NBPF20;NBPF10	glucose	Carbohydrate	Glycolysis, Gluconeogenesis, Pyruvate	-0.01	9.24E-07
cg19693031	TXNIP	3-methyl-2-oxobutyrate	Amino Acid	Leucine, Isoleucine, Valine	-0.02	2.14E-13
cg19693031	TXNIP	3-methyl-2-oxovalerate	Amino Acid	Leucine, Isoleucine, Valine	-0.01	1.23E-06
cg19693031	TXNIP	1-carboxyethylvaline	Amino Acid	Leucine, Isoleucine, Valine	-0.01	9.64E-06
cg19693031	TXNIP	mannonate*	Xenobiotics	Food Component/Plant	-0.01	1.61E-11
cg19693031	TXNIP	Gluconate^a^	Xenobiotics	Food Component/Plant	-0.01	4.83E-08
cg02988288	TXNIP;NBPF20;NBPF10	mannonate*	Xenobiotics	Food Component/Plant	-0.01	9.01E-06
cg19693031	TXNIP	1-palmitoyl-2-arachidonoyl-GPE (16:0/20:4)*	Lipid	Phosphatidylethanolamine (PE)	-0.01	4.31E-11
cg19693031	TXNIP	1-palmitoyl-2-oleoyl-GPE (16:0/18:1)	Lipid	Phosphatidylethanolamine (PE)	-0.01	4.03E-09
cg19693031	TXNIP	alanine	Amino Acid	Alanine, Aspartate	-0.01	9.68E-10
cg02988288	TXNIP;NBPF20;NBPF10	alanine	Amino Acid	Alanine, Aspartate	-0.01	3.02E-08
cg19693031	TXNIP	erythronate*	Carbohydrate	Aminosugar	-0.01	2.57E-08
cg19693031	TXNIP	Mannose^a^	Carbohydrate	Fructose, Mannose, Galactose	-0.01	9.30E-07
cg19693031	TXNIP	glutamine	Amino Acid	Glutamate	0.01	1.11E-06
cg19693031	TXNIP	2-hydroxybutyrate/2-hydroxyisobutyrate^a^	Amino Acid	Glutathione	-0.01	1.66E-10
cg19693031	TXNIP	ribitol	Carbohydrate	Pentose	-0.01	5.98E-06
cg19693031	TXNIP	1-carboxyethylphenylalanine	Amino Acid	Phenylalanine	-0.01	1.63E-06
cg19693031	TXNIP	X—24295	Unnamed	Unnamed	-0.01	7.53E-15
cg19693031	TXNIP	X—24334	Unnamed	Unnamed	-0.02	3.62E-12
cg19693031	TXNIP	X—12101	Unnamed	Unnamed	-0.01	4.17E-09
cg19693031	TXNIP	X—14056	Unnamed	Unnamed	-0.01	3.03E-08
cg19693031	TXNIP	X—19438	Unnamed	Unnamed	-0.01	1.32E-07
cg11969813	P4HB	1-palmitoyl-2-oleoyl-GPE (16:0/18:1)	Lipid	Phosphatidylethanolamine (PE)	0.01	1.23E-08
cg00683922	PFKFB2	1-carboxyethylphenylalanine	Amino Acid	Phenylalanine	0.01	3.57E-08
cg22699725	PFKFB2	alanine	Amino Acid	Alanine, Aspartate	0.01	3.31E-07
cg00683922	PFKFB2	alanine	Amino Acid	Alanine, Aspartate	0.01	4.54E-07
cg00683922	PFKFB2	erythronate*	Carbohydrate	Aminosugar	0.01	2.87E-06
cg00683922	PFKFB2	1-palmitoyl-2-oleoyl-GPE (16:0/18:1)	Lipid	Phosphatidylethanolamine (PE)	0.01	7.71E-07
cg00683922	PFKFB2	X—14,056	Unnamed	Unnamed	0.01	1.00E-05
cg06291107	BLCAP	mannonate*	Xenobiotics	Food Component/Plant	0.01	3.08E-07
cg06291107	BLCAP	glucose	Carbohydrate	Glycolysis, Gluconeogenesis, Pyruvate	0.01	5.76E-07
cg06291107	BLCAP	ribitol	Carbohydrate	Pentose	0.01	6.12E-07
cg06291107	BLCAP	1-palmitoyl-2-arachidonoyl-GPE (16:0/20:4)*	Lipid	Phosphatidylethanolamine (PE)	0.01	2.21E-07
cg06291107	BLCAP	1-palmitoyl-2-oleoyl-GPE (16:0/18:1)	Lipid	Phosphatidylethanolamine (PE)	0.01	8.49E-07
cg01676795	POR	1,5-anhydroglucitol (1,5-AG)	Carbohydrate	Glycolysis, Gluconeogenesis, Pyruvate	-0.01	2.25E-07
cg01676795	POR	glycosyl ceramide (d18:2/24:1, d18:1/24:2)*	Lipid	Hexosylceramides (HCER)	-0.01	8.92E-06
cg01676795	POR	methylsuccinoylcarnitine	Amino Acid	Leucine, Isoleucine, Valine	0.01	3.89E-06
cg00092123	KIAA1211L	mannonate*	Xenobiotics	Food Component/Plant	0.01	4.37E-07
cg09029192	TNRC6C	1-palmitoyl-2-oleoyl-GPE (16:0/18:1)	Lipid	Phosphatidylethanolamine (PE)	0.01	7.23E-07
cg09029192	TNRC6C	1-palmitoyl-2-arachidonoyl-GPE (16:0/20:4)*	Lipid	Phosphatidylethanolamine (PE)	0.01	2.36E-06
cg13547913	OCIAD1	1-palmitoyl-2-arachidonoyl-GPE (16:0/20:4)*	Lipid	Phosphatidylethanolamine (PE)	0.01	8.90E-07
cg13547913	OCIAD1	1-palmitoyl-2-oleoyl-GPE (16:0/18:1)	Lipid	Phosphatidylethanolamine (PE)	0.01	1.51E-06
cg18939666	PEF1	1-palmitoyl-2-oleoyl-GPE (16:0/18:1)	Lipid	Phosphatidylethanolamine (PE)	0.01	9.27E-07
cg18939666	PEF1	1-palmitoyl-2-arachidonoyl-GPE (16:0/20:4)*	Lipid	Phosphatidylethanolamine (PE)	0.01	3.02E-06
cg18939666	PEF1	mannonate*	Xenobiotics	Food Component/Plant	0.01	8.02E-06
cg10167677	THBS4	mannonate*	Xenobiotics	Food Component/Plant	0.01	1.06E-06
cg10167677	THBS4	methylsuccinoylcarnitine	Amino Acid	Leucine, Isoleucine, Valine	0.01	3.62E-06
cg12350057	PPP1R3E	erythronate*	Carbohydrate	Aminosugar	0.01	1.14E-06
cg00994936	DAZAP1	mannonate*	Xenobiotics	Food Component/Plant	0.01	1.72E-06
cg04326337	RPRD1B	ribitol	Carbohydrate	Pentose	0.01	1.73E-06
cg06721411	DQX1	ribitol	Carbohydrate	Pentose	0.01	2.10E-06
cg06721411	DQX1	1-palmitoyl-2-oleoyl-GPE (16:0/18:1)	Lipid	Phosphatidylethanolamine (PE)	0.01	4.59E-06
cg06721411	DQX1	1-palmitoyl-2-arachidonoyl-GPE (16:0/20:4)*	Lipid	Phosphatidylethanolamine (PE)	0.01	7.90E-06
cg27305772	MUS81	1-palmitoyl-2-oleoyl-GPE (16:0/18:1)	Lipid	Phosphatidylethanolamine (PE)	0.01	2.32E-06
cg09879165	CACNA2D2	1-palmitoyl-2-oleoyl-GPE (16:0/18:1)	Lipid	Phosphatidylethanolamine (PE)	0.00	3.24E-06
cg15007470	TCF3	1-palmitoyl-2-oleoyl-GPE (16:0/18:1)	Lipid	Phosphatidylethanolamine (PE)	0.01	6.22E-06
cg19707375	BAIAP2-AS1	glycosyl ceramide (d18:2/24:1, d18:1/24:2)*	Lipid	Hexosylceramides (HCER)	-0.01	9.55E-06
cg08088075		1-palmitoyl-2-arachidonoyl-GPE (16:0/20:4)*	Lipid	Phosphatidylethanolamine (PE)	0.01	1.73E-07
cg09777883		ribitol	Carbohydrate	Pentose	0.01	2.19E-07
cg08088075		1-palmitoyl-2-oleoyl-GPE (16:0/18:1)	Lipid	Phosphatidylethanolamine (PE)	0.01	2.54E-07
cg09777883		1-palmitoyl-2-oleoyl-GPE (16:0/18:1)	Lipid	Phosphatidylethanolamine (PE)	0.01	1.20E-06
cg09777883		1-palmitoyl-2-arachidonoyl-GPE (16:0/20:4)*	Lipid	Phosphatidylethanolamine (PE)	0.01	2.70E-06
cg22896572		ribitol	Carbohydrate	Pentose	0.01	5.38E-06

^a^ indicates associations previously reported in other studies. *indicates compounds that have not been officially confirmed based on a standard, but Metabolon is confident in its identity

TXNIP (mainly cg1969303), was associated with 25 metabolites including 1,5 anhydroglucitol and pyruvate from the glycolysis pathway, and metabolites from fructose/mannose metabolism, alanine and aspartate metabolism, aminosugars, pentose metabolism, phenylalanine metabolism, branched chain amino acids, glutamate metabolism, glutathione metabolism, phosphatidylethanolamines and metabolites classified as food components. Among the remaining 41 associations, were DQX1 (DEAQ Box Polypeptide 1) with phosphatidylethanolamines and ribitol, BLCAP (Bladder Cancer Associated Protein) with ribitol, mannonate, glucose, and phosphatidylethanolamines, DAZAP1 (Deleted In Azoospermia-Associated Protein 1) with mannonate, OCIAD1 (Ovarian Carcinoma Immunoreactive Antigen) with phosphatidylethanolamines, PFKFB2 (6-Phosphofructo-2-Kinase/Fructose-2,6-Biphosphatase 2) with 1-carboxyethylphenylalanine, alanine, erythronate and phosphatidylethanolamines, and POR (P450 Cytochrome Oxidoreductase) with 1,5 anhydroglucitol and a hexosylceramide among others.

### Multi-omics networks in T2D

**Fig. 7:**
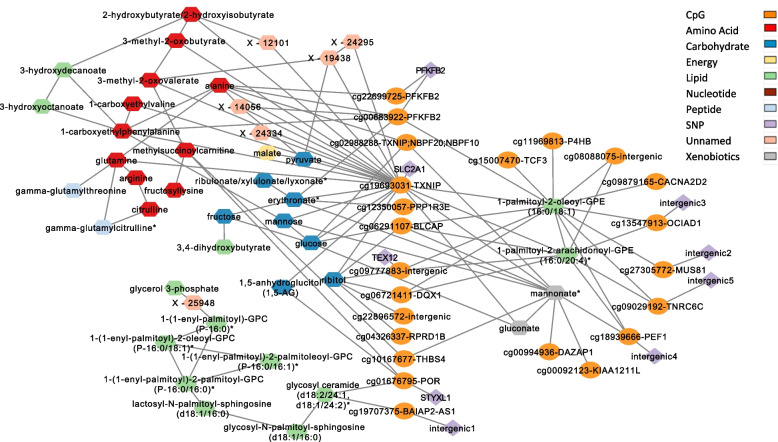
Multi-omics network of T2D methylated genes associations with metabolites, and their meQTLs integrated with metabolic networks. Oval nodes are CpGs, diamonds are SNPs, and hexagons are metabolites

A multi-omics network was constructed from all associations obtained and metabolic networks to identify pathways combining methylation and metabolism. Integrating the 66 CpG-metabolite pairs with 9 meQTL SNPs of the CpGs in those pairs and the T2D metabolites that had significant partial correlations with the metabolites involved in those 66 CpG-metabolite pairs, we obtained a network with 105 nodes (SNP/CpG/metabolite) and 165 edges (associations or correlations). The largest subnetwork had 75 nodes and 120 edges and harbored all CpGs that had metabolic associations (Fig. 7, Additional file 3: Figure S1).

The methylated genes interconnect together through three major pathways: amino acids (11 metabolites), carbohydrates (8 metabolites) and lipids (13 metabolites). Amino acids formed a subnetwork that linked TXNIP, PFKFB2, THBS4, and POR with alanine and aspartate metabolism, urea cycle metabolites, branched chain amino acid metabolites, glutathione metabolism, phenylalanine metabolism, and glutamate metabolism. The carbohydrates formed a subnetwork that linked TXNIP, PFKFB2, PPP1R3E, BLCAP, DQX1, RPRD1B, and POR to glycolysis metabolism (1,5 anhydroglucitol, glucose, pyruvate), fructose, mannose, pentose metabolism (ribitol, ribulonate), and the aminosugar erythronate. A group of 8 lipids formed a subnetwork of ceramides and sphingosines linked to POR and BAIAP2-AS1. Other major connectivity is through hub metabolites (with many edges to genes) as 1-palmitoyl-2-oleoyl-GPE (16:0/18:1) connection to 13 CpGs in 11 genes and mannonate’s (xenobiotic) connection to six genes.

To enrich the metabolic pathway information, the associations of metabolites with BMI, lipoprotein cholesterols and triglycerides were identified. Of the 43 T2D metabolites in this network, 16 showed higher significance in association with BMI, LDL-C, HDL-C or triglycerides compared to their association with T2D (Additional file 2: Table S11), among which 1-palmitoyl-2-oleoyl-GPE (16:0/18:1) was associated with triglycerides and linked to 13 CpGs.

## Discussion

This study used a large number of subjects from the Qatari population that has a high prevalence of diabetes and used the largest methylation EPIC array. Whole genome sequencing and metabolomics data were incorporated to enrich the identification of biological pathways associated with methylation and diabetes.

A total of 66 CpG sites were significantly associated with T2D, of which 63 CpGs were novel in the insulin signaling pathway, fructose and mannose pathway and other T2D relevant pathways. Twenty-two of the identified CpGs had meQTLs in 688 CpG-SNP associations, among which 130 were novel associations and 51 pairs were in novel loci. Novel nonsynonymous and 5’UTR variants were identified in T2D methylated sites of SERPINF1, DOCK10, and TXNIP. Several meQTLs SNPs had GWAS associations with T2D related traits, and causal relationships were statistically inferred between novel CpG sites in HK1 and PFKFB2 and HbA1c (*p* < 0.0001). Finally, 61 CpG-metabolite pairs out of all 66 identified pairs were novel, among which TXNIP, DAZAP1, BLCAP, and OCIAD1 were found associated with various carbohydrates, lipids, amino acids and xenobiotics. The constructed multi-omics network revealed several methylation-metabolism pathways that related to T2D risks or complications.

The most significantly-associated T2D methylated gene, thioredoxin-interacting protein (TXNIP), plays a role in insulin sensitivity, is a tumor suppressor that is upregulated in diabetes, is induced when glucose levels are elevated, and its deficiency improves glucose tolerance and increases insulin sensitivity in high fat diet-induced obesity [34, 39]. TXNIP suppresses glucose uptake by directly binding and suppressing the glucose transporter, GLUT1 (SLC2A1), by facilitating its clathrin-mediated endocytosis [34, 40]. When elevated, TXNIP induces β-cell apoptosis, while its deficiency protects against type I and type II diabetes by promoting β-cell survival [41, 42]. It is also known to be involved in kidney injury and with IL-1 $\upbeta$ in the pathogenesis of T2D [43, 44]. Thus, findings from the meQTL analysis, linking SLC2A1 to TXNIP and its several metabolic associations support and add to those known functions of the gene. Besides TXNIP, pathway analysis and the known biology of the novel methylated genes support their relevance to T2D. For example, PPP1R3E, PRKAR2A and HK1 are involved in insulin signaling and resistance (Additional file 2: Table S5). Together with the several T2D-related pathways reported for HK1, further evidence of its relevance to T2D has been provided by GWAS associations of HK1 genetic variants with HbA1c [45], with the strongest signals reported in [46]. HK1 is in the family of hexokinases, which are known to phosphorylate glucose to produce glucose-6-phosphate, the first step in most glucose metabolism pathways [47]. Its association with the insulin signaling pathway and hyperinsulinemia [48], along with being part of the carbohydrate absorption, glycolysis, fructose and mannose metabolism pathways further emphasizes the statistically inferred causality relation. Other methylated genes have been associated with T2D incidence [49] and cardiovascular disease [50]. For example, CACNA2D2 is involved in pathways related to T2D-associated coronary heart disease, cardiomyopathy, cardiac muscle contraction [51]. Variants of DOCK6 have been associated with GWAS studies on coronary heart disease and total and HDL cholesterol levels [52, 53]. THBS4 has been associated with more advanced peripheral artery disease and T2D [54]. It has also been reported to mediate breast cancer inflammation and growth in mouse models in response to hyperglycemia and TGF-beta [55]. IDO2, one of the isoforms of IDO (Indoleamine 2, 3-dioxygenase) which forms part of the tryptophan metabolism and catalyzes the conversion of tryptophan to kynurenine, is upregulated in T2D patients with baseline tryptophan being associated with higher risk of incident T2D [56].Variants of PBRM1 have been reported to be associated with BMI-related traits and diabetes pathways [57, 58]. While the pathways and biological function of the identified genes could partially be explained from previous findings, our study reveals other biological functions through linking methylation with genomic variants and metabolic pathways.

The meQTL analysis indicated the effect of genomic variation on methylation levels of 22 T2D CpGs, whereas the remaining 44 T2D CpGs are more likely affected by environment/lifestyle. The association of the missense SNP rs1136287 in the SERPINF1/SMYD4 locus with cg11692409 aligns with the previously reported GWAS association of SMYD4 with T2D that is harbored in the SRR locus [4]. The DOCK10 association with diabetic atherosclerosis [33] and its gene expression association with glucose in pancreatic islets [59] suggest that the nonsynonymous meQTL SNPs in DOCK10 may link the gene’s methylation to T2D genetic factors, diabetes complications or mechanisms in islet cells. The presence of a 5’UTR variant in SLC2A1 that codes the GLUT1 protein and that controls methylation in TXNIP is another novel finding that confirms previous biological links found between TXNIP and SLC2A1 [34, 35, 40] as discussed above.

GWAS associations of meQTL SNPs with T2D-related traits such as HbA1c (HK1 and PFKFB2), BMI (EFEMP2, CFL1), eGFR and creatinine (PFKFB2), diabetic nephropathy, urate levels (EFEMP2), bilirubin (HK1), and white blood cell counts (DOCK10, EFEMP2, SLC2A1, CFL1, PFKFB2) [51, 60, 61], further emphasize the involvement of the meQTL genes in the pathogenesis of T2D. In other words, SNP variation may alter methylation levels in T2D CpGs inducing various biological perturbations and complications in T2D patients. Furthermore, the mendelian randomization analysis suggested a causal relationship of DNA methylation in HK1 with HbA1c levels, supporting previous findings of HK1’s GWAS associations with HbA1c and HK1 pathways involving glucose [47], insulin signaling and hyperinsulinemia [48].

This study reports novel T2D methylation-metabolite associations. Compared to a few previously reported metabolic associations with TXNIP CpG cg19693031, this study reveals a much larger role of TXNIP in T2D metabolic pathways that span several pathways of interest, including branched chain amino acids, alanine, lipid metabolism, and sugars among others. DQX1, previously identified to be methylated with T2D in an Arab population, has no meQTL associated with it, suggesting that the metabolic associations of DQX1 with lipid metabolism and ribitol could be largely affected by lifestyle factors. Metabolic associations with genes known to associate with bladder cancer (BLCAP), ovarian cancer (OCIAD1), and infertility caused by azoospermia (DAZAP1) could highlight the importance of integrating both methylation and metabolomics for assessing T2D complications.

Integrating methylation with both genomics and metabolomics has an important role in understanding the cause-effect direction of methylation-metabolism in T2D, i.e., whether methylation drives metabolic changes through gene expression or changes in metabolites cause changes in methylation. For example, methylation of TXNIP is controlled by SNPs in SLC2A1, known to be involved in glucose metabolism, and thus TXNIP association with the glycolysis pathway could be through this meQTL. Similarly, we showed that the meQTL of PFKFB2 had SNPs associated with HbA1c, eGFR, and creatinine, and at the same time the gene had associations with carbohydrates, lipids, phenylalanine and alanine that are involved in both T2D [17, 18] and kidney function [62, 63], suggesting that the metabolic perturbations could be an end effect of the meQTL. Linking methylated genes to metabolism may help future investigation of the population-specificity of some methylated genes to Qataris. Perturbation of the methylation-metabolic pathway by changing lifestyle factors or nutrition in Qataris may result in different methylation patterns in Qataris compared to the other populations and thus may help explain T2D mechanisms in Qataris.

Multi-omics networks have facilitated the identification of metabolic pathways linked to the methylated genes, thus revealing their functionality through metabolites that were associated with T2D risks/complications. One example is the alanine subnetwork that links PFKFB2 and TXNIP to the urea cycle and to amino acids, where amino acids were associated with triglycerides (alanine association with triglycerides: discovery *p*-value of *p* = 6.4 × 10^–12^, replication *p*-value of *p* = 9.3 × 10^–13^) and where both urea cycle and alanine have been associated with kidney failure [64]. Moreover, 1-carboxyethylphenylalanine in the alanine subnetwork was associated with triglycerides (*p*-value discovery: 1.42 × 10^–34^, *p*-value replication: 4.6 × 10^–21^) and is known to be associated with hypertension [65]. Both links extend the GWAS findings for PFKFB2 described above and reveal the involvement of triglycerides in T2D methylation. The association of 1-palmitoyl-2-oleoyl-GPE(16:0/18:1) with triglycerides (discovery *p*-value *p* = 6.47 × 10^–100^, replication *p*-value *p* = 1.39 × 10^–80^) and its link to 11 methylated genes suggests a possible cause of methylation linked to a diabetes risk/complication that is common between those genes. Mannonate, a xenobiotic (food component/plant) is another example that shares a network with six genes, namely TXNIP, BLCAP, THBS4, PEF1, DAZAP1, and KIAA1211L and may suggest a possible xenobiotic effect on methylation. The networks also highlighted a *STYXL1–POR–sphingosines/ceramides–LDL,HDL–steroids* connection, where the unique association of POR with a lipid subnetwork of sphingosines, GPCs, and ceramides is supported by the fact that gene’s production of the enzyme cytochrome P450 oxidoreductase is required for the synthesis of cholesterol and steroid hormones (https://medlineplus.gov/genetics/gene/por/, and references therein). Moreover, since reproductive and fertility complications may arise from diabetes [66], it is interesting to note that STYXL1, the meQTL gene of the cg01676795 (POR) is known to be associated with seminal vesicle tumor and male reproductive organ benign neoplasm [67], that may help in understanding the link of POR to a network of sphingosines and ceramides which play a role in forming steroids [68].

Limitations of the study include measuring methylation from whole blood rather than pancreatic islets as that was the only available tissue from the Qatar Bio Bank. Another limitation was the inability to correct for medication as that information was not yet annotated by the provider at the time of the study.

## Conclusions

A total of 66 CpG sites were identified to be associated with T2D, of which 63 were novel and linked to biological pathways of T2D. Genomic drivers of the CpG sites were identified in 688 significant CpG-SNP pairs, among which 181 CpG-SNP pairs were novel associations or in novel loci. Several GWAS associations in meQTLs’ genes revealed various underlying factors in pathways linked to T2D. The study identified novel nonsynonymous and 5’UTR variants associated with T2D methylation in SERPINF1, DOCK10, and TXNIP, as well as the statistically-inferred causal relation between HbA1c and each of the HK1 and PFKFB2 methylation sites. A total of 61 novel methylation-metabolite associations revealed association of several methylated genes with T2D metabolic pathways including population specific genes such as DQX1, among others. The multi-omics network suggested a number of T2D biological mechanisms associated with methylation, including the association of triglyceride metabolism and xenobiotics to 11 and 6 methylated genes respectively, indicating a common factor among those methylated genes and may have a role in their methylation. The pathways identified linked to steroid metabolism, and to metabolites associated with BMI, lipoprotein cholesterols and kidney function, namely STYXL1-POR, SLC2A1-TXNIP, and PFKFB2, may reveal the association of T2D risk factors or complications with methylation mechanisms. Finally, this study revealed several novel methylated genes related to T2D, with their associated genomic variants and metabolic pathways.

### Supplementary Information

**Additional file 1.** Methods.

**Additional file 2:** **Table S1.** Results of two way discovery replication T2D EWAS using cohorts 1 and 2. **Table S2.** Results of lookup of the identified 66 T2D CpGs in databases and previous literature. **Table S3.** Replication of literature findings. **Table S4.** eQTM at FDR < 0.05 from BIOS QTL site for all 66 T2D CpGs. **Table S5.** KEGG pathway (Pathways relevant to diabetes or complications are highlighted). **Table S6.** All 688 CpG-SNP significant associations in 27 loci. **Table S7.** GWAS associations for the SNPs in the neighborhood of meQTL SNPs as reported in the GWAS catalogue. **Table S8.** GWAS associations for the SNPs in the neighborhood of meQTL SNPs as reported in the Stanford bio bank. **Table S9.** A)Discovery and Replication cohorts for T2D MWAS, B)T2D significant metabolites with the discovery and replication statistics. **Table S10.** 77 CpG-metabolite association results from meta-analysis (11 colored rows are associations with high heterogeneity (*p*<0.05)). **Table S11.** Associations of 16 metabolites from the multi-omics network with BMI, lipoproteins and triglycerides. **Table S12.** Characteristics of metabolomics samples used for the T2D MWAS.

**Additional file 3: Figure S1.** Full omics network of CpG-metabolites and associated SNP-CpGs and associated metabolite-metabolite pairs.

## Data Availability

All data is provided in the manuscript and in the supplementary tables.

## Abbreviations

CpG: *5'—C—phosphate—G—3'*, Cytosine and guanine separated by a phosphate group
EWAS: Epigenome Wide Association Study
GWAS: Genome Wide Association Study
QBB: Qatar Bio Bank
QTL: Quantitative Trait Loci
meQTL: methylation Quantitative Trait Loci
SNP: Single Nucleotide Polymorphism
T2D: Type 2 Diabetes
MWAS: Metabolome Wide Association Study
