# Supplementary material for: Integrated epigenome, whole genome sequence and metabolome analyses identify novel multi-omics pathways in type 2 diabetes: a Middle Eastern study
Source: BMC Med. 2023 Sep 8;21:347. doi: 10.1186/s12916-023-03027-x (PMC10485955; doi:10.1186/s12916-023-03027-x)
Supplement: Supplementary file 1 — Additional file 1. Methods. [file 12916_2023_3027_MOESM1_ESM.docx]

Additional methods:

A total of 1056 blood samples were collected according to the QBB protocol, and DNA was extracted and kept in freezers and aliquoted at a concentration of 550 ng per sample. Samples were quantified using Qubit dsDNA HS Assay Kit (ThermoFisher Scientific, USA). DNA bisulfite conversion was performed using EZ-96 DNA Methylation Kit (Zymo Research, USA) according to the manufacturer’s protocol.  The bisulfite converted DNA was processed with the Infinium EPIC Methylation Kit (Illumina, USA) following the manufacturer’s protocol guide.  The stained beadchips were scanned on the Illumina iScan system. After quality control, 102 samples remained (41% with T2D) for analysis.

For cohort 3, older subjects were selected with an average age of 53 and 55 years for diabetics and non-diabetics respectively. All samples had data for T2D, BMI, gender, age, HbA1c, neutrophils, basophils, eosinophils, lymphocytes and leukocytes, WBC, creatinine and uric acid.

Using BMI as a covariate may over-adjust the CpG association with T2D given the strong correlation between T2D and BMI. However, cohort 1 had a higher BMI difference between those with and without T2D compared to cohort 2, and thus finding associations with T2D with and without BMI adjustment were used. Together with the two way discovery replication, this approach enables finding commonly significant CpGs between the two cohorts.

Quality control on whole genome data was done to remove SNPs with minor allele frequency <0.05, remove SNPs that violated Hardy Weinberg equilibrium (p < 10^-6^), and SNPs with a genotype call rate <98%.

Manhattan plots for meQTLs and EWAS results were created using qqman ([https://joss.theoj.org/papers/10.21105/joss.00731#](https://joss.theoj.org/papers/10.21105/joss.00731)). Principal component analysis was computed using “plink” software and components were included as covariates in the association analyses.
