# Supplementary material for: Integrated epigenome, whole genome sequence and metabolome analyses identify novel multi-omics pathways in type 2 diabetes: a Middle Eastern study
Source: BMC Med. 2023 Sep 8;21:347. doi: 10.1186/s12916-023-03027-x (PMC10485955; doi:10.1186/s12916-023-03027-x)
Supplement: Supplementary file 3 — Additional file 3: Figure S1. Full omics network of CpG-metabolites and associated SNP-CpGs and associated metabolite-metabolite pairs. [file 12916_2023_3027_MOESM3_ESM.pdf]

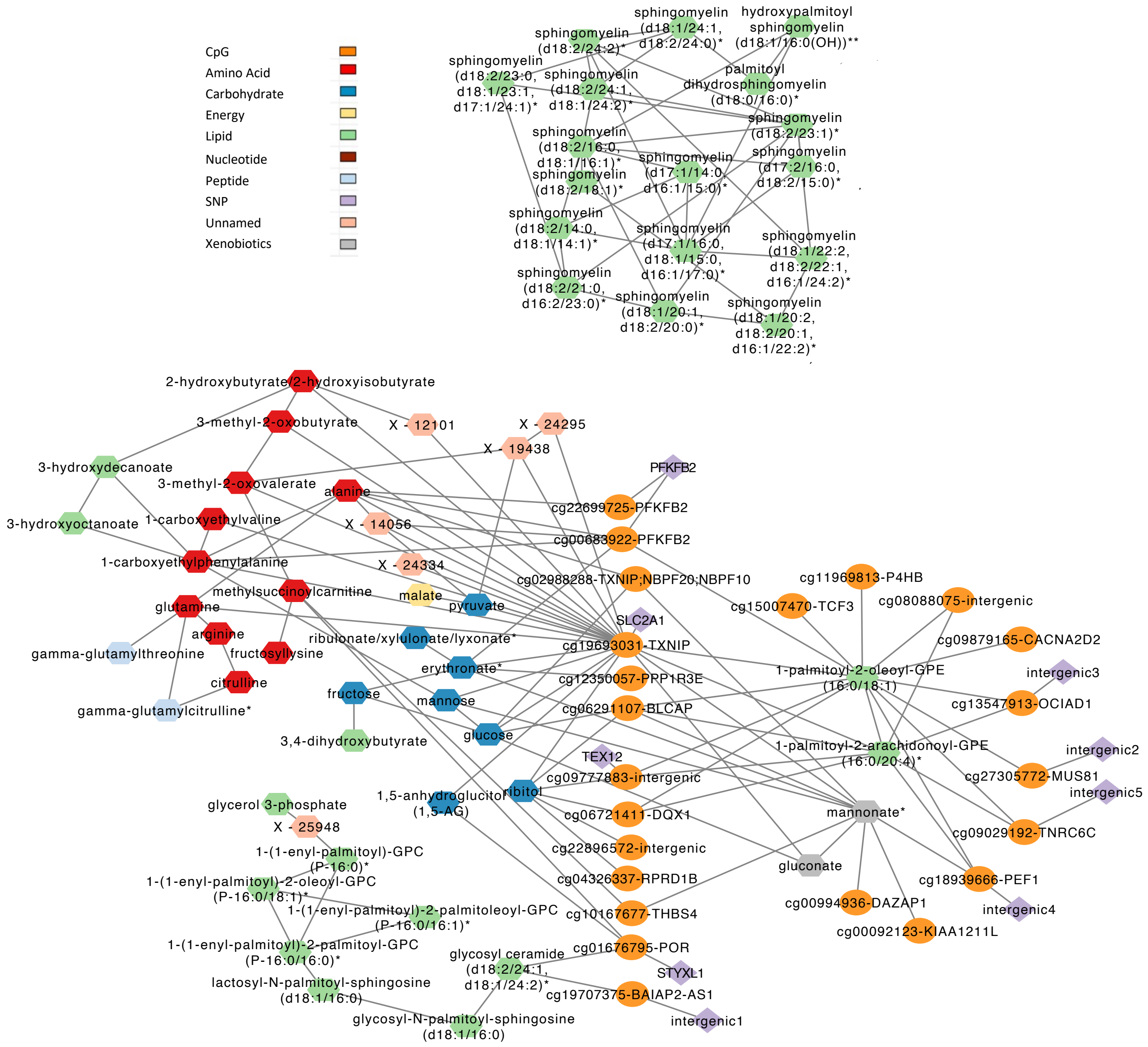

Figure S1: Full multi-omics network of T2D methylated genes associations with metabolites, and their meQTLs integrated with metabolic networks. Oval nodes are CpGs, diamonds are SNPs, hexagons are metabolites.
